# Bidirectional Associations Between Activity Diversity and Sleep

**DOI:** 10.1093/geroni/igaa057.2248

**Published:** 2020-12-16

**Authors:** Soomi Lee, Susan Charles, David Almeida

**Affiliations:** 1 University of South Florida, Tampa, Florida, United States; 2 BSS, Irvine, California, United States; 3 Pennsylvania State University, University Park, Pennsylvania, United States

## Abstract

Activity diversity is important for psychological well-being and cognitive functioning. Yet, little is known about the relationship between activity diversity and sleep. This study examined how overall and nightly sleep health are associated with activity diversity. Participants (N=1841) from the Midlife in the United States Study II provided activity data for 8 days. We constructed overall and daily activity diversity scores. A composite score of overall sleep health across 8 dimensions and nightly sleep duration were measured. Analyses adjusted for sociodemographics, total activity time, and positive/negative affect. Participants with poorer sleep health overall had a lower activity diversity. On days following nights with short (<6hrs) or long (>8hrs) sleep duration, participants engaged in fewer-than-usual activities. Conversely, fewer daily activities also predicted long (but not short) sleep duration. Our results suggest cyclical associations between poor sleep health and activity diversity day-to-day, which, may accumulate over time to form a bidirectional relationship.

